# Suppression of Rho-associated kinase 1 (ROCK1) promotes human hematopoietic stem cell expansion by attenuating mitochondrial fission

**DOI:** 10.1038/s41375-025-02770-9

**Published:** 2025-09-16

**Authors:** Xuepeng Wang, Baskar Ramdas, Ramesh Kumar, Ji Zhang, Reuben Kapur

**Affiliations:** 1 Department of Pediatrics, Herman B Wells Center for Pediatric Research, Indiana University School of Medicine, Indianapolis, IN USA; 2 Indiana University Melvin and Bren Simon Comprehensive Cancer Center, Indianapolis, IN USA

**Keywords:** Haematopoietic stem cells, Haematopoietic stem cells

Hematopoietic stem cells (HSCs) are remarkable cells that sustain lifelong hematopoiesis through their unique abilities to self-renew and generate all mature blood lineages. Harnessing these properties is fundamental for therapeutic applications such as hematopoietic stem cell transplantation (HSCT) and gene therapies for hematologic disorders. However, effectively expanding HSCs ex vivo remains a major challenge, particularly because the proliferation required for their expansion often induces cellular stress that compromises their long-term repopulating capacity, in part by perturbing mitochondrial dynamics [1, 2]. During cell division, mitochondria naturally undergo cycles of fission to ensure proper distribution into daughter cells [3]; however, excessive mitochondrial fission is a known driver of reactive oxygen species (ROS) accumulation and apoptosis, both of which impair HSC self-renewal and function [4–6]. Notably, HSCs within the native bone marrow (BM) niche reside in a physiologically hypoxic microenvironment that supports quiescence and balanced proliferation, preserving self-renewal capacity [7, 8]. Understanding the molecular characteristics of this protective niche is critical for designing strategies to maintain HSC functionality during ex vivo expansion. Despite substantial efforts, current methods remain limited in their ability to recapitulate the supportive signals of the BM niche in culture, underscoring the need for novel approaches to preserve HSC function.

To elucidate mechanisms that support HSC homeostasis in vivo, we performed single-cell CITE-sequencing on mouse BM-derived HSCs processed under physiological hypoxia (5% O₂) versus standard normoxia (21% O₂). Our analysis revealed that Rho-associated kinase 1 (Rock1), a serine/threonine kinase central to cytoskeletal dynamics, apoptosis regulation, and mitochondrial homeostasis [6, 9], was markedly downregulated in hypoxia-collected HSCs compared to normoxia-processed controls (Fig. 1A). Moreover, several upstream regulators of ROCK1, including Rho family GTPases and their guanine nucleotide exchange factors (GEFs), were also downregulated under hypoxia (Fig. 1A), while multiple Rho pathway negative regulators were upregulated (Fig. 1B). Consistently, downstream targets of ROCK1 involved in cytoskeletal remodeling and apoptosis showed reduced expression in hypoxic HSCs (Fig. 1C). Given that the BM microenvironment is intrinsically hypoxic, these coordinated changes in hypoxic HSCs suggest that suppression of ROCK1 signaling is a molecular hallmark of the in vivo homeostatic state sustaining HSC maintenance.

**Fig. 1:**
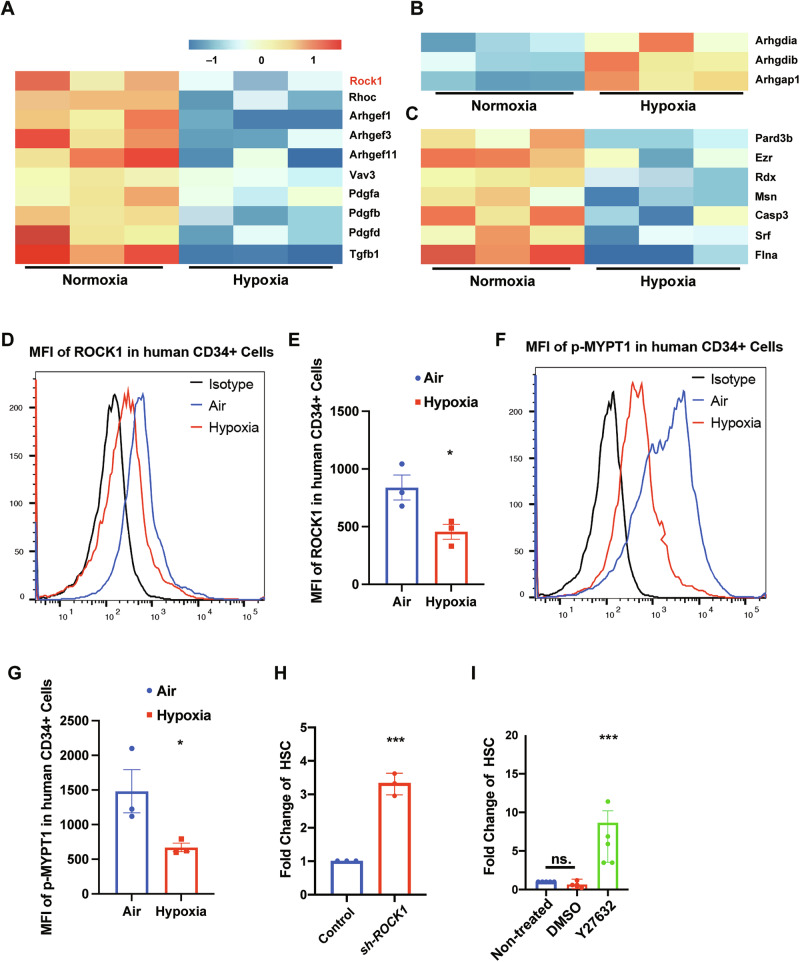
Hypoxia suppresses ROCK1 expression and activity in mouse HSCs and human CD34^+^ cells, enhancing phenotypic HSC expansion upon ROCK1 inhibition. **A** Heatmap showing single-cell CITE-seq expression profiles of Rock1 and its upstream Rho GTPases and GEFs in mouse BM HSCs processed under normoxia (21% O₂) versus physiological hypoxia (5% O₂). **B** Expression of Rho pathway negative regulators increased under hypoxia, as revealed by single-cell analysis. **C** Downstream Rock1 targets involved in cytoskeletal remodeling and apoptosis exhibit reduced expression in hypoxic HSCs. **D**, **E** Intracellular flow cytometry confirms decreased ROCK1 protein levels in hypoxia-processed human CD34⁺ cells (*n* = 3). **F** Representative histograms of p-MYPT1 staining. **G** Quantification of p-MYPT1 mean fluorescence intensity (MFI, *n* = 3). **H**, **I** Quantification of absolute numbers of phenotypic HSCs in cultures treated with *shROCK1* (*n* = 3) or Y-27632 (*n* = 5) compared to controls. Data shown as mean ± s.e.m. *p < 0.05; **p < 0.01; ***p < 0.001 by one-way ANOVA.

Given the observed ROCK1 downregulation under hypoxic conditions in mouse HSCs, we next determined whether this change is conserved in human HSCs and whether it contributes causally to the maintenance of human HSC properties. We therefore hypothesized that suppressing ROCK1 activity during ex vivo culture might mimic key protective aspects of the BM niche, preserving human HSC function during expansion. To test this hypothesis, we transplanted cord blood-derived human CD34⁺ cells into NSG mice. After 2 months, we harvested BM and purified human CD34⁺ cells under hypoxic conditions to assess their phenotypic and functional characteristics. Quantitative real-time PCR and intracellular flow cytometry revealed that both ROCK1 mRNA and protein levels were significantly reduced under hypoxia compared to normoxia (Figs. 1D, E and S1A). Furthermore, phosphorylation of MYPT1—a canonical ROCK1 substrate—was decreased under hypoxic conditions in human CD34^+^ cells [10], indicating reduced ROCK1 kinase activity (Fig. 1F, G). These data establish that low ROCK1 expression and activity are conserved features of HSCs maintained under physiological hypoxia.

To explore the functional consequences of ROCK1 suppression in ex vivo expansion of human HSCs, we used both genetic and pharmacological inhibition approaches. Lentiviral shRNA targeting ROCK1 efficiently knocked down ROCK1 in human CB CD34⁺ cells, as confirmed by RT-qPCR (Fig. S1B, C). In parallel, pharmacologic inhibition with Y-27632, a specific ROCK inhibitor used at concentrations of 2.5–5 μM to minimize off-target effects. Both strategies led to significant expansion of phenotypically defined HSCs (Lin⁻CD34⁺CD38⁻CD45RA⁻ CD49f⁺CD90⁺) over 4 days of culture after ROCK1 inhibition in serum-free medium supplemented with SCF, TPO, and FLT3-L (Fig. 1H, I). Notably, ROCK1 suppression not only increased numbers of phenotypic HSCs but also elevated total cell numbers, suggesting enhanced proliferation without apparent loss of primitive stem cell markers (Fig. S1D, E).

Mechanistically, ROCK1 inhibition by using shRNA or Y-27632 in human CD34^+^ cells exhibited reduced mitochondrial ROS levels, as assessed by MitoSOX staining (Fig. 2A–C), alongside decreased mitochondrial mass (Fig. S2A–C) and membrane potential measured using MitoTracker and JC1 staining, respectively (Fig. S2D–F). These findings indicate that ROCK1 inhibition suppresses mitochondrial activity and mitoROS generation, both of which are critical contributors to HSC exhaustion during ex vivo expansion. Since excessive mitochondrial fission is a key driver of ROS accumulation, we next investigated whether ROCK1 suppression affects mitochondrial dynamics by regulating the GTPase DRP1, a central mediator of mitochondrial fission [4].

**Fig. 2:**
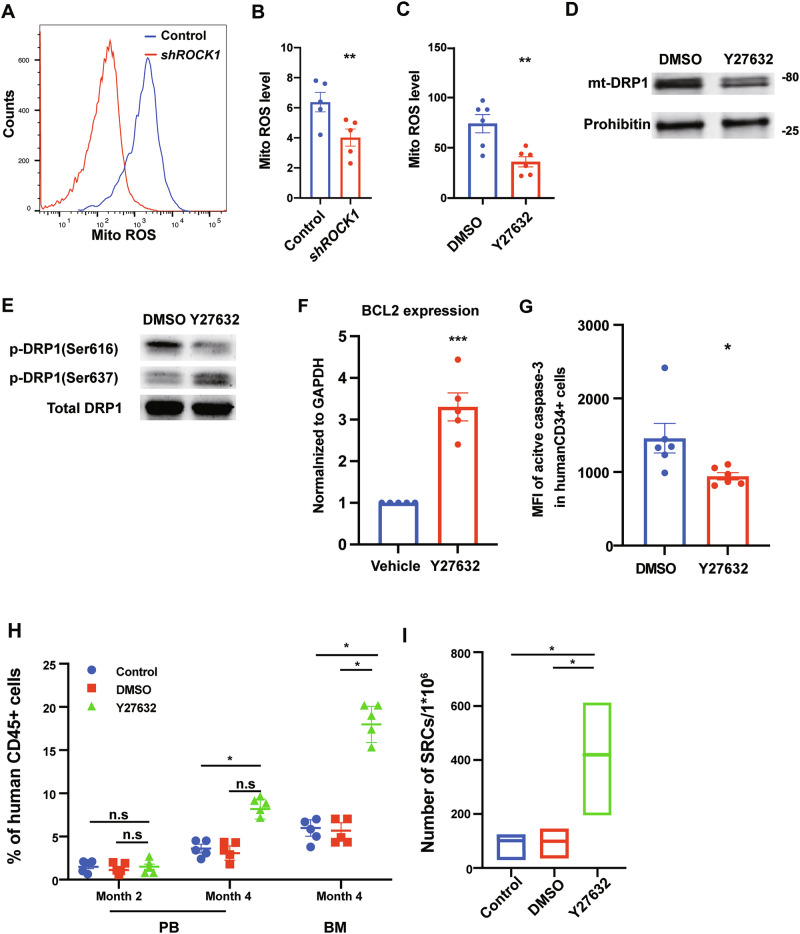
ROCK1 inhibition reduces DRP1-mediated mitochondrial fission, decreases mitochondrial ROS and apoptosis, and enhances functional engraftment of human HSCs. **A** Representative histograms of MitoSOX staining showing reduced mitochondrial ROS in *shROCK1* transduced human CD34⁺ cells versus controls. **B** Quantification of MitoSOX MFI in *shROCK1* transduced human CD34⁺ cells versus controls (*n* = 5). **C** Quantification of MitoSOX MFI in Y-27632 or DMSO-treated human CD34⁺ cells (*n* = 5). **D** Western blot illustrating reduced DRP1 mitochondrial localization upon ROCK1 inhibition. **E** Western blot analysis of DRP1 phosphorylation at Ser616 and Ser637 in DMSO versus Y-27632-treated human CD34^+^ cells. **F** Quantitative RT-PCR showing increased BCL2 expression in Y-27632-treated human CD34^+^ cells (*n* = 5). **G** Intracellular flow cytometry showing decreased active Caspase-3 levels in Y-27632-treated human CD34^+^ cells (*n* = 5). **H** Percentage of human CD45^+^/mouse CD45^−^ cells in PB and BM at 2 months and 4 months after transplantation in NSG mice with non-treated control, DMSO, or Y-27632-treated human CD34^+^ cells (*n* = 5). **I** HSC frequencies (line in the box) and confidence intervals (box) presented as numbers of SRCs in 1 × 10^6^ human CD34+ cells. Data shown as mean ± s.e.m. *p < 0.05; **p < 0.01; ***p < 0.001 by one-way ANOVA.

Indeed, ROCK1 inhibition led to a marked decrease in DRP1 mitochondrial localization (Fig. 2D), supporting the idea that ROCK1 modulates mitochondrial fission via DRP1 upstream of ROS production. Given that DRP1-mediated fission promotes apoptosis, and its activity is tightly regulated by phosphorylation at key residues, we examined DRP1 phosphorylation status. We found that Y-27632 treatment significantly reduced phosphorylation at DRP1 Ser616, a site associated with DRP1 mitochondrial translocation and mitochondrial fission, while concurrently increasing phosphorylation at DRP1 Ser637, which is known to inhibit DRP1 activity [11, 12](Fig. 2E). Intracellular staining confirmed the reduction of p-DRP1(Ser616) levels in Y-27632-treated cells compared to controls (Fig. S3A, B). Consistent with attenuated DRP1 mitochondrial localization, ROCK1-inhibited cells exhibited increased expression of the anti-apoptotic gene BCL2 and reduced levels of active Caspase-3, as demonstrated by RT-PCR and intracellular flow cytometry (Figs. 2F, G and S3C). Together, these results suggest that ROCK1 suppression prevents excessive mitochondrial fragmentation by modulating DRP1 phosphorylation—reducing p-DRP1(Ser616) and enhancing p-DRP1(Ser637)—thereby limiting DRP1 mitochondrial localization, decreasing ROS accumulation, and reducing apoptosis to preserve mitochondrial and cellular integrity during HSC proliferation.

Recognizing that phenotypic characterization alone does not fully reflect functional HSC potential, we next assessed whether ROCK1 inhibition enhances ex vivo expansion of HSCs with durable engraftment capacity. We performed limiting dilution assays by transplanting Y-27632-treated human CB CD34⁺ cells into sublethally irradiated NOD-SCID IL-2rγnull (NSG) mice. These assays demonstrated significantly improved engraftment of treated cells in both peripheral blood at two and 4 months, as well as in BM at 4 months post-transplantation (Fig. 2H). Calculations of SCID-repopulating cell (SRC) frequencies revealed approximately a fourfold increase in functional HSCs following Y-27632 treatment compared to controls (Figs. 2I and S3D) (*p* < 0.05). Importantly, secondary transplantation confirmed that ROCK1-inhibited HSCs retained robust long-term self-renewal capacity, with threefold higher chimerism rates in recipient mice (Fig. S3E) (*p* < 0.001), and our findings extend previous work demonstrating that inhibition of RhoA GTPase activity enhances HSC proliferation and engraftment in murine models by providing mechanistic insights into how suppression of its downstream effector ROCK1 regulates mitochondrial dynamics and preserves human HSC function during ex vivo expansion [13].

These findings are consistent with previous work showing that ROCK inhibition reduces apoptosis in other stem cell types, such as pluripotent and mesenchymal stem cells, by limiting cytoskeletal tension and mitochondrial stress [14]. Taken together, these results delineate a mechanistic pathway by which ROCK1 suppression enhances HSC expansion, and extend these insights by providing direct evidence that ROCK1 is a pivotal regulator coupling mitochondrial dynamics to ROS-mediated apoptotic pathways during ex vivo proliferation of human HSCs. Collectively, our data support a model in which HSCs in their native hypoxic BM niche maintain low ROCK1 expression. This appears to be part of a homeostatic program minimizing excessive mitochondrial fission and ROS accumulation, thereby preserving self-renewal capacity. By transiently inhibiting ROCK1 ex vivo, we can mimic key aspects of this protective niche, enhancing the expansion of phenotypically and functionally defined human HSCs with reduced oxidative stress and apoptosis.

In summary, our study establishes that suppression of ROCK1—either genetically or pharmacologically—reduces DRP1-mediated mitochondrial fission, lowers mitochondrial ROS, limits apoptosis, and promotes ex vivo expansion of functional human HSCs. By elucidating the mechanistic link between ROCK1 activity and mitochondrial homeostasis, our findings provide a framework for optimizing HSC expansion protocols. While our findings highlight the potential of ROCK1 inhibition for ex vivo HSC expansion, it is important to consider the broader context of ROCK-targeting strategies. Pharmacologic inhibitors such as Y-27632 and fasudil have been widely used in preclinical studies and, in the case of fasudil, in clinical settings for vascular indications [9]. However, these compounds can affect multiple ROCK isoforms and, at high concentrations, may lead to off-target effects. Moreover, in vivo studies have demonstrated that systemic or long-term inhibition of ROCK activity can disrupt tissue homeostasis, particularly in rapidly renewing compartments such as the hematopoietic and gastrointestinal systems [15]. In contrast, our study confines ROCK1 inhibition to a brief ex vivo window during HSC culture, thereby limiting toxicity while effectively enhancing expansion and function. These results suggest that transient modulation of ROCK1 activity is a viable and potentially translatable approach to optimize HSC-based therapies, particularly for settings where stem cell dose remains limiting. Future studies should explore the timing and dosage of ROCK1 inhibition, assess long-term genomic stability and multilineage differentiation, and evaluate potential clinical applications to improve outcomes in HSCT and gene therapy.

## Supplementary information

Supplementary Methods

Supplemental Figure 1

Supplemental Figure 2

Supplemental Figure 3

## Data Availability

The datasets generated during and/or analyzed during the current study are available from the corresponding author on reasonable request.
