# Publisher Correction: Suppression of Rho-associated kinase 1 (ROCK1) promotes human hematopoietic stem cell expansion by attenuating mitochondrial fission

**DOI:** 10.1038/s41375-025-02786-1

**Published:** 2025-10-13

**Authors:** Xuepeng Wang, Baskar Ramdas, Ramesh Kumar, Ji Zhang, Reuben Kapur

**Affiliations:** 1 Department of Pediatrics, Herman B Wells Center for Pediatric Research, Indiana University School of Medicine, Indianapolis, IN USA; 2 Indiana University Melvin and Bren Simon Comprehensive Cancer Center, Indianapolis, IN USA

**Keywords:** Haematopoietic stem cells, Haematopoietic stem cells

Correction to: *Leukemia* 10.1038/s41375-025-02770-9, published online 16 September 2025

Following the publication of this article, it was noted that a number of formatting and grammatical errors had been missed during typesetting. These errors have now been corrected. These corrections have no impact on the findings of the article. The original article has been corrected.

