# Supplementary material for: Suppression of Rho-associated kinase 1 (ROCK1) promotes human hematopoietic stem cell expansion by attenuating mitochondrial fission
Source: Leukemia. 2025 Sep 16;39(11):2825–9. doi: 10.1038/s41375-025-02770-9 (PMC12531631; doi:10.1038/s41375-025-02770-9)
Supplement: Supplementary file 1 — Supplementary Methods [file 41375_2025_2770_MOESM1_ESM.docx]

**Supplementary Methods**

**Mice**

Immunodeficient 6- to 8-week-old female NSG (NOD.Cg-Prkdc^scid^ IL2rg^tm1Wjl^/SzJ) mice were bred by the In Vivo Therapeutics Core at Indiana University School of Medicine (IUSM), supported in part by the NIDDK Cooperative Centers of Excellence in Hematology (CCEH). Mice were maintained under specific pathogen-free conditions in the Laboratory Animal Resource Center (LARC) at IUSM. All animal experiments were conducted in accordance with protocols approved by the Institutional Animal Care and Use Committee (IACUC) of IUSM.

**Human CD34^+^ CB cell collection and culture**

Umbilical cord blood (CB) units were obtained from HumanCells Biosciences (Milpitas, CA, USA). All studies were approved by the Institutional Review Board of IUSM. Mononuclear cells were isolated by density gradient centrifugation, followed by CD34⁺ cell enrichment using an immunomagnetic selection kit (Miltenyi Biotec, 130-046-702). The purity of isolated human CD34⁺ CB cells exceeded 90%. Cells were cultured in StemSpan II serum-free medium (STEMCELL Technologies, 09650) supplemented with 100 ng/mL stem cell factor (SCF), 100 ng/mL Fms-like tyrosine kinase 3 ligand (Flt3L), and 100 ng/mL thrombopoietin (TPO).

For Y-27632 treatment, the small molecule inhibitor (Cayman Chemical, 10005583) was added at the initiation of culture at a final concentration of 2.5–5 μM, adjusted based on donor variability. Cultures were maintained in a humidified incubator at 37°C with 5% O₂. Cells were harvested after four days for downstream gene expression and flow cytometry analyses.

**RNA extraction and real-time PCR**

Cells were harvested by fluorescence-activated cell sorting (FACS), and total RNA was extracted using the RNeasy Mini Kit (Qiagen, 74106) according to the manufacturer’s instructions. Reverse transcription was performed using the Superscript III kit (Thermo Fisher Scientific, 18080093). Quantitative real-time PCR (qPCR) reactions were conducted using SYBR Green PCR Master Mix (Thermo Fisher Scientific) on an Agilent Mx3000P qPCR System. GAPDH was used as the housekeeping gene for normalization. Relative mRNA expression levels were calculated using the ΔΔCt method, with control samples set as baseline (normalized to 1).

**Vector construction and virus production**

For shRNA vector construction, single-stranded oligonucleotides were synthesized by Genewiz (South Plainfield, NJ, USA). After annealing, double-stranded oligonucleotides were ligated into the shRNA expression plasmid using T4 DNA ligase (New England Biolabs, M2622L). Successfully constructed vectors were verified by Sanger sequencing (Genewiz). Lentiviruses were produced by transfecting 293FT cells with the validated shRNA plasmids, and supernatants containing lentiviral particles were collected 48 hours post-transfection. A non-targeting shRNA vector expressing GFP was used as a control in all experiments.

**Lentivirus transduction of human CD34^+^ cells**

Lentiviral particles were concentrated using 30% polyethylene glycol 8000 (PEG8000; Sigma, 1546605) precipitation. Prior to transduction, freshly isolated human CD34⁺ cells were pre-stimulated for 24 hours in StemSpan II medium supplemented with SCF (100 ng/mL), Flt3L (100 ng/mL), TPO (100 ng/mL), and cyclosporin H (CsH; 20 μM). Lentiviruses were added to the same medium at a multiplicity of infection (MOI) of 50–200, followed by an 8-hour incubation. This transduction step was repeated three times. After transduction, cells were cultured in fresh StemSpan II medium supplemented with SCF, Flt3L, and TPO, and allowed to recover for two days before proceeding to in vitro or in vivo assays.

**Flow cytometry and sorting**

Human CD34^+^ cells were sorted for different phenotypes by using the following antibodies. Mouse anti-human antibodies were used to detect CD34 (APC, BD Biosciences, 560940), CD45RA (Taxe-red, BD Biosciences, 562298), CD90 (BV421, BD Biosciences, 562556), CD49f (cy5.5, BD Biosciences, 562495), CD38 (PE, BD Biosciences, 560981), Lin (FITC, Biolegend, 348807)，Lin (FITC, BD Biosciences, 340546). CD3 (BV421, BD Biosciences, 562427), CD33 (PE, BD Biosciences, 555450), CD19 (PE, BD Biosciences, 555413) and CD45 (APC, BD Biosciences, 555485). Cells were analyzed on a BD FACS-Canto flow cytometer (BD Biosciences), and cell sorting (under low sheath fluid pressure) was conducted on a BD FACSAria cell sorter (BD Biosciences, Franklin Lakes, NJ, USA).

For intracellular staining, cells were fixed and permeabilized using the Cytofix/Cytoperm Fixation/Permeabilization Kit (BD Biosciences, 554714) according to the manufacturer’s protocol. Cells were then stained with primary antibodies against phospho-DRP1 (Ser616) (Cell Signaling Technology, 3455), phospho-MYPT1 (Thr696) (Cell Signaling Technology, 5163), and active Caspase-3 (FITC-conjugated, BD Biosciences, 550480) for 30 minutes at room temperature. After washing, cells were analyzed on a BD FACS Canto flow cytometer. Data were processed using FlowJo software (BD Biosciences).

**MitoSOX, MitoTracker, and JC-1 Staining**

Live human CD34⁺ cells were first stained with cell surface markers as described above. After washing, cells were incubated with MitoSOX™ Red Mitochondrial Superoxide Indicator, MitoTracker Green FM, or JC-1 dye (all from Thermo Fisher Scientific) according to the manufacturer’s protocols. Briefly, cells were incubated with each mitochondrial dye for 15 minutes at 37°C in a humidified incubator. Following staining, cells were washed with ice-cold PBS by centrifugation at 300 × g for 10 minutes at 4°C. The supernatant was removed, and cells were gently vortexed and resuspended in 500 μL PBS. Samples were immediately analyzed on a BD LSRFortessa flow cytometer (BD Biosciences).

**Limiting dilution analysis (LDA)**

The frequency of human SCID-repopulating cells (SRCs) was determined by LDA as previously described^1^. Increasing doses of human CD34⁺ cells (1000, 3,000, or 10,000 cells) were intravenously injected into sublethally irradiated NSG recipient mice (350 cGy from a ^137Cs source, single dose). Four months post-transplantation, human engraftment was assessed by flow cytometry of peripheral blood and bone marrow samples, quantifying human CD45⁺, mouse CD45^−^ cells. For long-term engraftment assays evaluating HSC self-renewal, 3 × 10⁶ bone marrow cells harvested from primary recipients in the 10,000-cell group were intravenously transplanted into secondary sublethally irradiated NSG recipient mice.

**Statistical analysis**

Data were analyzed using GraphPad Prism (GraphPad Software, San Diego, CA, USA) and Microsoft Excel. Results are presented as mean ± standard deviation (SD) for normally distributed data, or mean ± standard error of the mean (s.e.m.) as specified in the figure legends. Statistical comparisons among more than two groups were performed using one-way ANOVA with appropriate post hoc tests. Differences were considered statistically significant at P < 0.05. Where applicable, relative risks were expressed as odds ratios with 95% confidence intervals. For comparisons between two measurement methods, the Bland & Altman approach was applied as recommended by Leukemia’s statistical guidelines^2^.

**References**

1. Doulatov S, Notta F, Laurenti E, Dick JE. Hematopoiesis: a human perspective. *Cell Stem Cell* 2012; 10**:** 120-136.
2. Bland JM, Altman DG. Statistical methods for assessing agreement between two methods of clinical measurement. *Lancet*. 1986 Feb 8;1(8476):307–310.

**Supplementary Figure Legends**

**Figure S1. Validation of ROCK1 knockdown and effects on phenotypic HSC expansion.**

(A) Quantitative RT-PCR showing ROCK1 and MYPT1 expression in hypoxia collected and processed human CD34^+^ cells (n=3). (B) The experimental strategy for virus transduction and transplantation of human CD34^+^ cells. (C) RT-qPCR confirming ROCK1 knockdown efficiency in transduced human CD34⁺ cells (n=5). (D) Quantification of total cell numbers after 4 days of culture with or without *shROCK1* transfection (n=3). (E) Quantification of total cell numbers after 4 days of culture with or without Y27632 treatment (n=5). Data shown as mean ± s.e.m. *p < 0.05; **p < 0.01; ***p < 0.001 by one-way ANOVA.

**Figure S2. ROCK1 inhibition reduces mitochondrial mass and membrane potential in human CD34⁺ cells.**

(A) Representative flow cytometry histograms of MitoTracker Green staining for mitochondrial mass in *shROCK1* or GFP (Control) transduced human CD34^+^ cells. (B) Quantification of mitochondrial mass tracker MFI in *shROCK1* or GFP (Control) transduced human CD34^+^ cells (n=5). (C) Quantification of mitochondrial mass tracker MFI in Y-27632 or DMSO treated human CD34^+^ cells (n=5). (D) Representative JC-1 red/green ratio plots demonstrating altered mitochondrial membrane potential. (E) and (F) Quantitative analyses of mitochondrial membrane potential metrics in human CD34^+^ cells following ROCK1 inhibition by using *shROCK1* or Y27632. Data shown as mean ± s.e.m. *p < 0.05; **p < 0.01; ***p < 0.001 by one-way ANOVA.

**Figure S3. ROCK1 suppression attenuates apoptosis markers and improves functional HSC readouts.**

(A) and (B) Intracellular flow cytometry showing reduced levels of phosphorylated DRP1(Ser616) in Y-27632-treated human CD34⁺ cells (n=3). (C) Flow cytometry quantification of active Caspase-3 expression in Y27632 treated human CD34⁺ cells. (D) Poisson statistical analysis. (n = 30). Solid lines represent the best-fit linear model for each group, dotted lines indicate 95% confidence intervals. (E) Percentage of human CD45^+^, mouse CD45^−^ cells in BM at 4 months in secondary recipients (n=5). Data shown as mean ± s.e.m. *p < 0.05; **p < 0.01; ***p < 0.001 by one-way ANOVA.
