# Supplementary figures and images for: Suppression of Rho-associated kinase 1 (ROCK1) promotes human hematopoietic stem cell expansion by attenuating mitochondrial fission
Source: Leukemia. 2025 Sep 16;39(11):2825–9. doi: 10.1038/s41375-025-02770-9 (PMC12531631; doi:10.1038/s41375-025-02770-9)

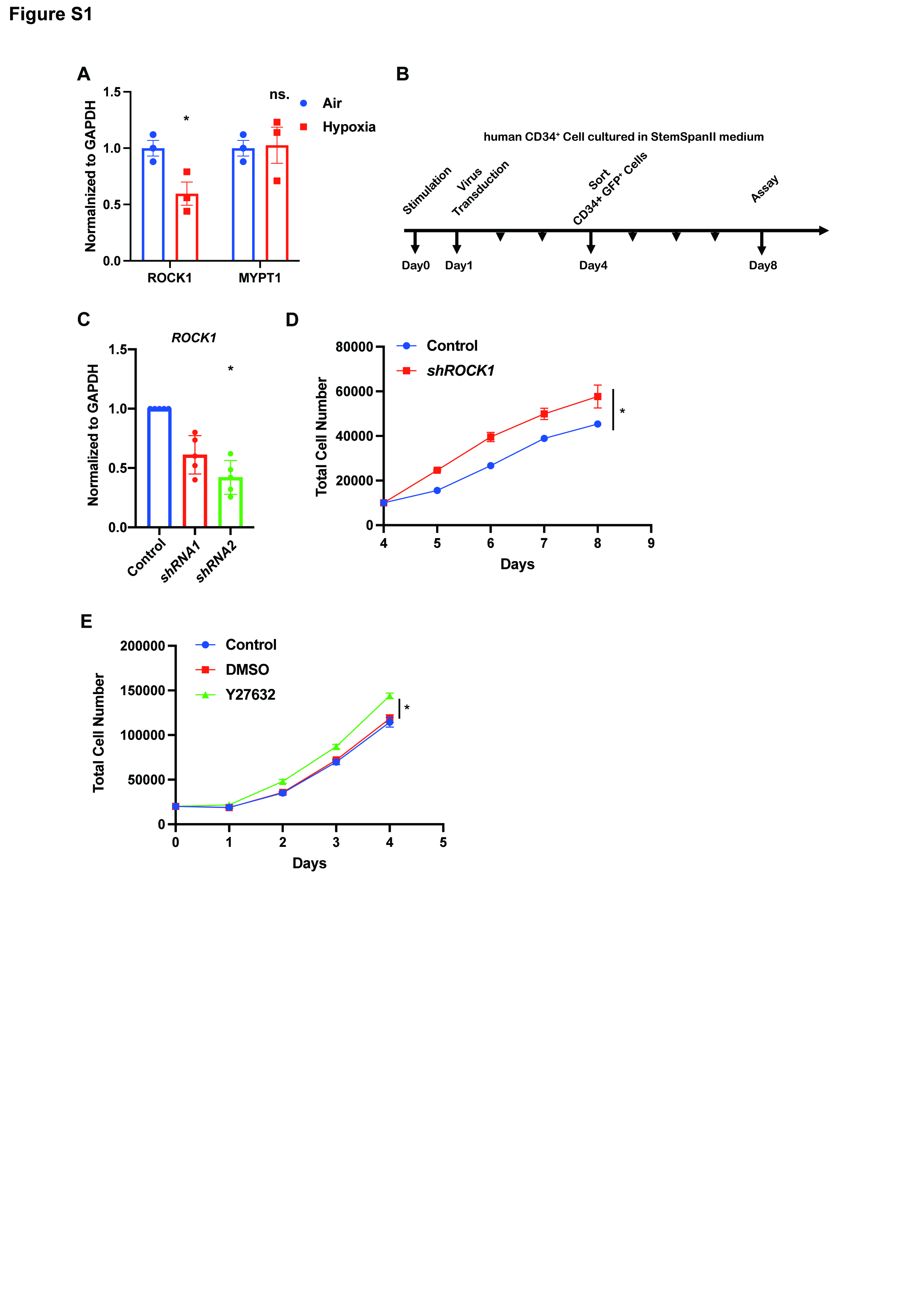

Supplement: Supplementary file 2 — Supplemental Figure 1 [file 41375_2025_2770_MOESM2_ESM.tif]

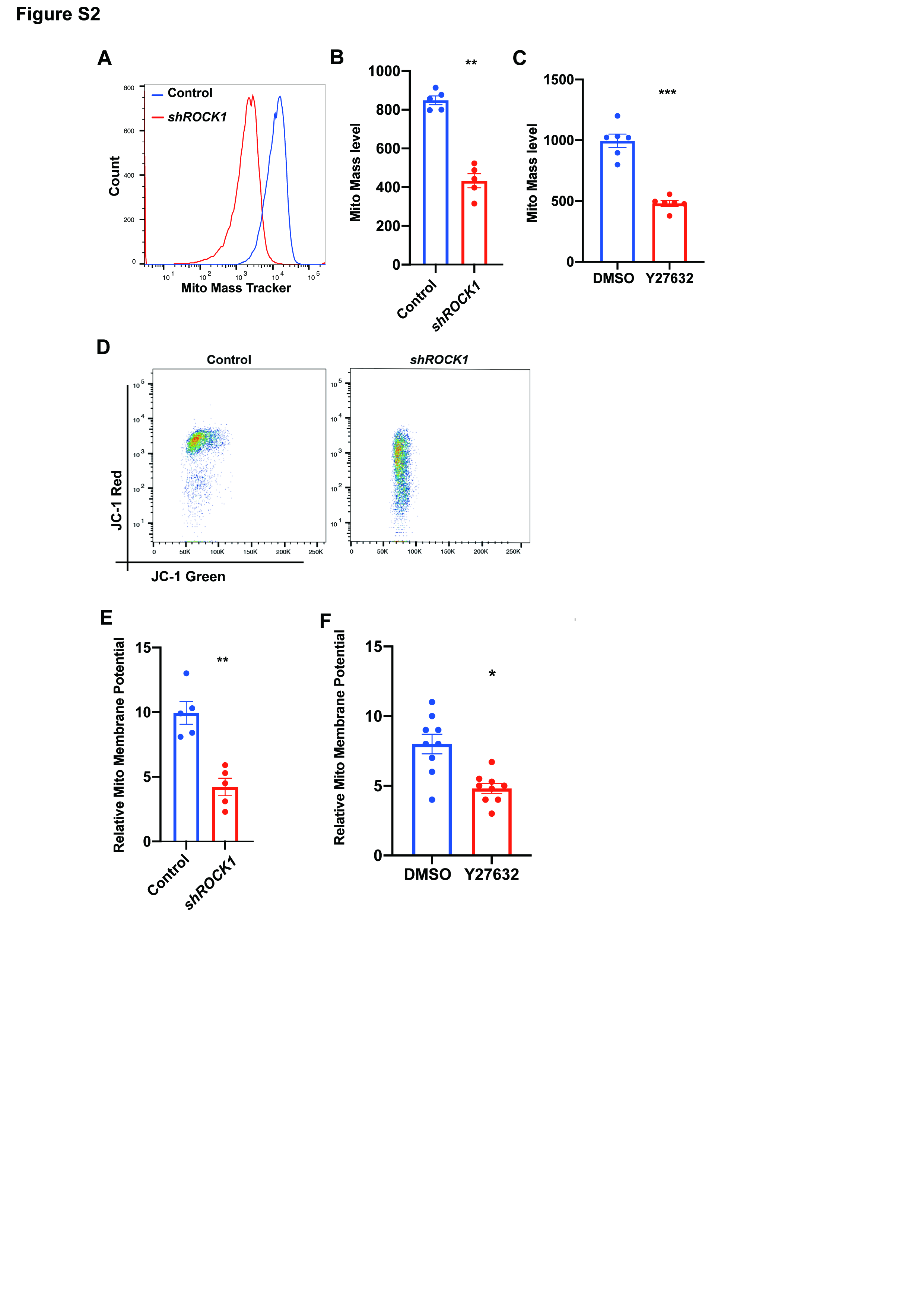

Supplement: Supplementary file 3 — Supplemental Figure 2 [file 41375_2025_2770_MOESM3_ESM.tif]

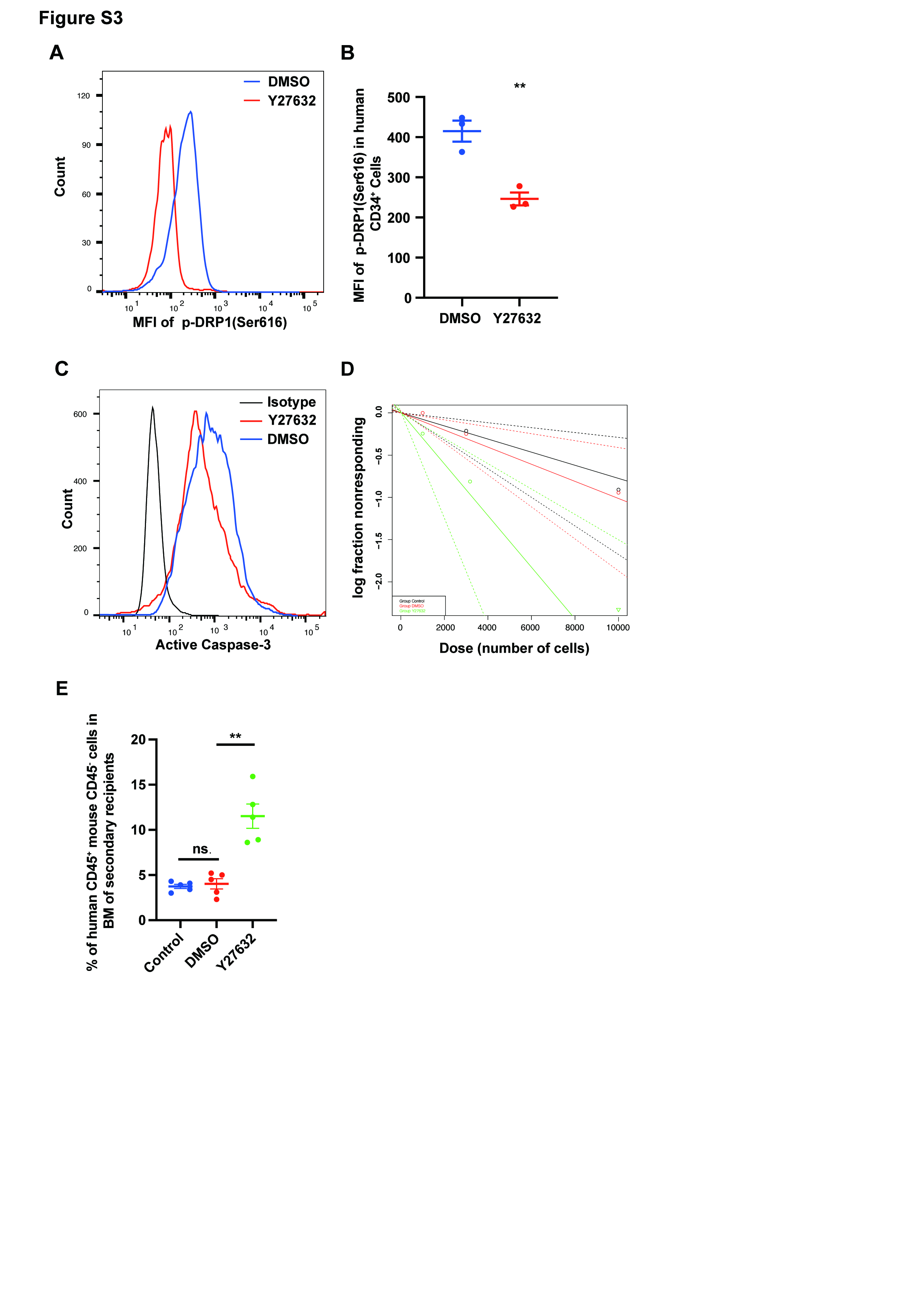

Supplement: Supplementary file 4 — Supplemental Figure 3 [file 41375_2025_2770_MOESM4_ESM.tif]
